# Interactive, Browser-Based Graphics to Visualize Complex Data in Education of Biomedical Sciences for Veterinary Students

**DOI:** 10.1007/s40670-022-01613-x

**Published:** 2022-09-22

**Authors:** Pamela Liebig, Heike Pröhl, Nadine Sudhaus-Jörn, Julia Hankel, Christian Visscher, Klaus Jung

**Affiliations:** 1 Institute for Animal Breeding and Genetics, University of Veterinary Medicine Hannover, Foundation, Bünteweg 17p, 30559 Hannover, Germany; 2 Institute of Zoology, University of Veterinary Medicine Hannover, Foundation, Hannover, Germany; 3 Institute of Food Quality and Food Safety, University of Veterinary Medicine Hannover, Foundation, Hannover, Germany; 4 Institute for Animal Nutrition, University of Veterinary Medicine Hannover, Foundation, Hannover, Germany

**Keywords:** Interactive graphics, Dynamic visualization, R Shiny, Biomedical education, Web-based learning

## Abstract

In veterinary education, data from biomedical or natural sciences are mostly presented in the form of static or animated graphics with no or little amount of interactivity. These kinds of presentations are, however, often not sufficient to depict the complexity of the data or the presented topic. Interactive graphics, which allow to dynamically change data and related graphics, have rarely been considered as teaching tool in higher education of biomedical disciplines for veterinary education so far. In order to study the applicability and the usefulness of interactive graphics in biomedical disciplines for lecturers and students in veterinary education, three different courses from biomedical disciplines were exemplarily implemented as interactive graphics and evaluated in a pilot study by a survey amongst lecturers and students of our university. The interactive graphics were built using the Shiny environment, a web-based application framework for the statistic software R. The survey amongst lecturers and students was based on questionnaires covering questions on the handling and usefulness of the digital teaching tools. In total, *n* = 327 students and *n* = 5 lecturers participated in the evaluation study which revealed that the interactive graphics are easy to handle for lecturers and students, and that they can increase the motivation for either teaching or learning. In total, 71% of the students affirmed that interactive graphics led to an increased interest for the presented contents and 76% expressed the wish to get taught more topics with interactive graphics. We also provide a workflow that can be used as a guideline to develop interactive graphics.

**Supplementary Information:**

The online version contains supplementary material available at 10.1007/s40670-022-01613-x.

## Introduction

The studies of veterinary medicine comprise several data-intensive subjects from natural sciences, anatomy and physiology with references to a large number of eukaryotic, prokaryotic and viral species. Research data in the form of simple tables, but also much more complex data such as sensor data [1], high-throughput sequencing and other omics data [2, 3], time series, or video and acoustic signals [4, 5] are involved in veterinary education as well. Modern approaches for the visualization of such complex data may be helpful for lecturers and students in teaching and learning the related study contents.

Within the educational context, computer visualizations are increasingly being used as part of learning material to depict scientific phenomena. Especially in multimedia learning environments, computer visualizations are becoming prevalent [6]. Computer-assisted learning has paved the way for transforming education at universities and has become an important part in medical education [7, 8]. In consequence, universities worldwide have extensively incorporated e-learning into their curriculum [9], offering students time and place flexibility [10]. In medical education, e-Learning has also become an integral part of the curriculum [11]. This includes lecture recordings and instructional videos as part of blended learning environments [12], where face-to-face teaching is combined with technology-based modalities. Furthermore, case-based e-Learning systems [13], simulations [14] and virtual reality tools [15] can help students of biological or medical disciplines to overcome the gap between theoretical knowledge and clinical applications.

At our veterinary school, besides courses for learning practical skills, most theoretical study contents are taught in the form of frontal teaching using wall-projected presentations. By screening a selection of PowerPoint files from different lecturers at our school, we found that individual slides often remain static and data presentations without interactive elements, although multimedia elements such as videos or audio material can be included in such documents. Digital teaching material such as video lectures, a heart sound library, and virtual microscope can provide the opportunity to deepen and complement the knowledge acquired in the lecture. However, most of those materials are not modifiable by the students and therefore rarely encourage students to question or reason the shown information. In contrast, interactive graphics can be manipulated in several ways and encourage students to participate actively in the learning process. This is of particular interest, since it is well known that active inclusion of students is important for learning motivation and long-term retention of information [16].

A large collection of browser-based simulations for topics from natural sciences is provided by the PhET environment [17]. These simulations also contain interactive elements, however, they do not cover specific topics from veterinary medicine such as animal nutrition or food science. Holzinger et al. [18] also presented interactive simulation software, which focuses only on human genetics.

Since 2013, the Shiny package, a web-based framework for the statistics software R, allows to integrate interactive graphics into a website [19]. In biomedical education of veterinary students, data from experiments and observations are often displayed as graphs that are used as teaching material for students. These graphs are often related to mathematical formulas and models. Interactive regulators such as sliders, click boxes or select lists can be used to change parameters of the models, and these changes are displayed in real time and can give the user a better understanding of how a specific parameter is affecting the outcome. This interactivity can invite students to explore complex data and dynamic processes. Fawcett [20] introduced interactive Shiny apps to incorporate research-informed learning and teaching into statistic courses, concluding that the method benefited the students in terms of their confidence in their understanding. In a similar direction, Williams and Williams and Potter et al. used R Shiny apps to enhance learning experience of statistic students [21, 22]. Previously such interactive dynamics visualizations were done with other programming languages such as HTML or Flash. These languages are, however, not widespread amongst academic staff in medicine. In contrast, R is widely used in science to do a variety of data analyses and thus is much more common amongst academic staff. With basic R skills, Shiny apps can be customized to specific topics. Due to the web-based framework, these apps can be displayed independently of hardware and software, which makes them easy to integrate in lectures. However, Shiny apps have rarely been used in the context of veterinary education so far.

Currently, there is little experience in veterinary education how to implement and integrate interactive graphics into the courses. Therefore, as part of the digitalization strategy of the State Lower Saxony (Germany), we implemented several teaching contents from the curriculum of veterinarians as interactive graphics. More precisely, three interactive graphics were implemented into different lectures from biomedical disciplines: zoology, animal nutrition and food science. Moreover, we conducted a pilot study in the form of a survey amongst lecturers and students who worked with these new tools. The aim of our survey was to evaluate the handling, usefulness and acceptance of interactive graphics amongst veterinary students and lecturers, and to derive a workflow that can be used as a road map to identify relevant topics and implement additional interactive graphics for veterinary education. The results of this pilot study are also considered as a basis for a larger study on the didactic effect of interactive graphics in veterinary education. The functionalities and didactic aims of the implemented graphics as well as the design of the pilot study are described in the “Methods” section, followed by the presentation of results and a discussion.

## Methods

### Local Setting

The author’s university is a specialized institution to educate and train veterinarians. The education consists of scientific-theoretical as well as practical components. The teaching and examination of the scientific-theoretical components are divided into three stages: first and second preclinical examination, including basic natural science such as physics, zoology, chemistry. The last stage, the veterinary state examination, consists of subjects like food science, animal nutrition and pathology. These subjects are taught in the form of compulsory lectures, seminars, laboratory courses and others. For our pilot study, we selected exemplarily three courses which are typically taught in the form of lectures and seminars, as a starting point to integrate interactive graphics into the veterinary education of our university. The first module was integrated in the zoology lecture; the latter two modules were animal nutrition and food science. We describe the implementation methods as well as the individual contents of each module in the following. The three modules were primarily designed to support the lecturer during the course, but they can also be used by the students for learning after the course within a web browser.

### Shiny Environment for Implementation of Interactive Graphics

We implemented the interactive graphics using the Shiny environment (Version 1.4.0) [19] based on the statistic software R (R Core Team [23]). This environment converts R scripts into user-friendly, visually appealing Shiny Applications, which allow users to display interactive graphics in the web browser. Regulator elements can be used to change the data basis behind the graphics. Besides interactive graphics, interactive tables can be integrated, too.

### Implemented Modules for Selected Courses

#### Zoology Module: the Effect of the pH on the Haemoglobin Oxygen Affinity Curve

The implemented graphic for this module provides the lecturer an environment to explain the pH effect on the oxygen affinity of blood haemoglobin step by step (Supplementary Fig. 1). When invoking the interactive graphic, the oxygen-haemoglobin dissociation curve is displayed for a physiological pH environment. By selecting a decrease or increase of the blood pH level or of the oxygen affinity, the lecturer can choose whether to acidify or alkalize the environment, which in return leads to changes in the dissociation curve. The changes in haemoglobin oxygen affinity can now be discussed with the students. For further interactive teaching, different explanations that clarify the shift of oxygen affinity are implemented. Thus, the lecturer can first discuss possible answers with the students and then select the answers from a selection list. Furthermore, users may obtain additional information about oxygen carriers by clicking on a second tab located on the top of the application.

#### Animal Nutrition: A Tool to Design a Diet for Bladder Stone Prophylaxis

**Fig. 1:**
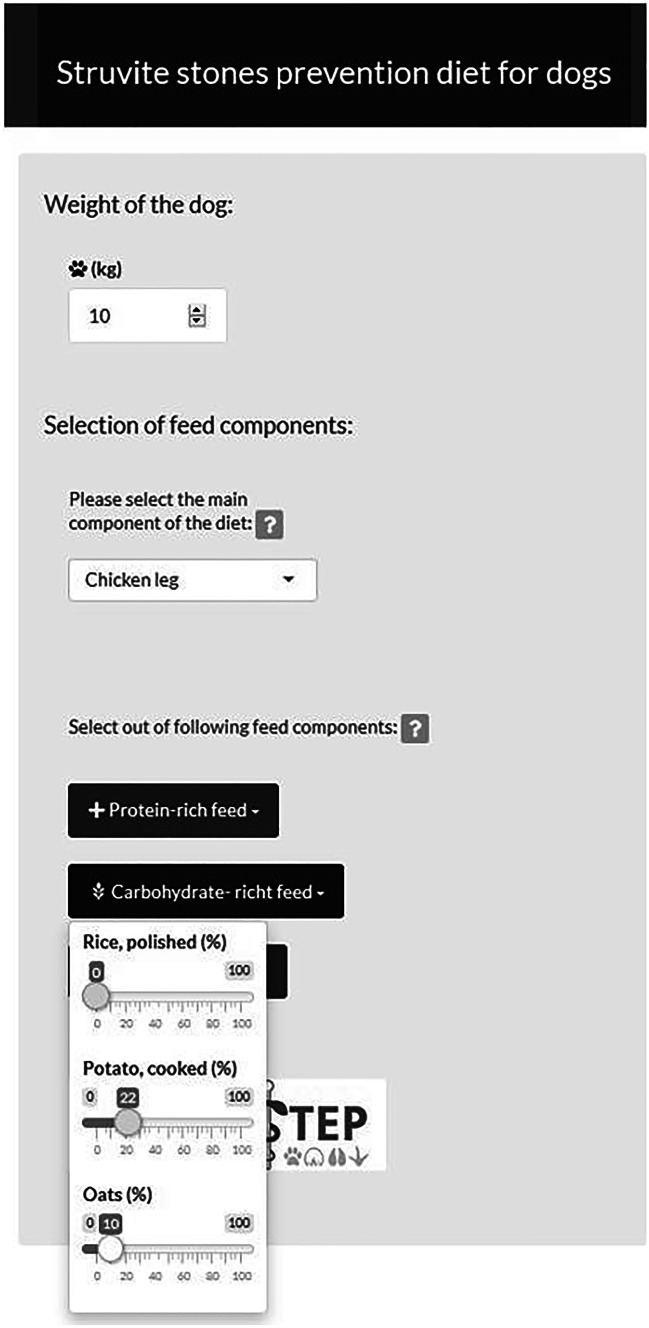
Screenshot of the user panel of the interactive teaching tool for the animal nutrition. The lecturer can use numeric input, selection list and sliders to demonstrate the effect of a created diet for dogs on mineral supply. A German version was used in the evaluation study

This web tool allows users to design a specific diet for bladder stone prophylaxis in dogs. The aim of this interactive graphic is to impart students the influence of the chemical and mineral composition specific to certain feed materials on the urine pH level and struvite stone formation. Users of this tool choose out of a selection of feed materials and one complementary feed as well. They decide which ingredients to add and the amount in percentage of those components within the ration. At first, users select a main component, which will be the primary ingredient in the diet. This main component covers 100% of the dog’s daily energy requirement. Afterwards, users can decide via sliders how many percent of other ingredients to add to the diet, by activating the sliders (Fig. 1).

**Fig. 2:**
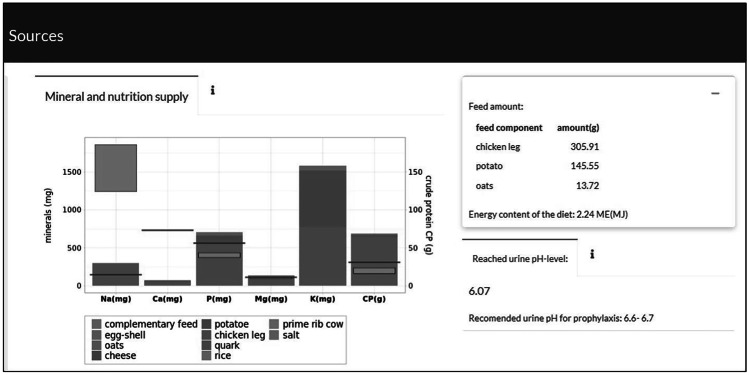
Screenshot of the bar plot indicating relevant components in struvite stone formation after selecting the feed components. Additionally, a table displays the amount of feed components, the energy content and the estimated urine pH level

**Fig. 3:**
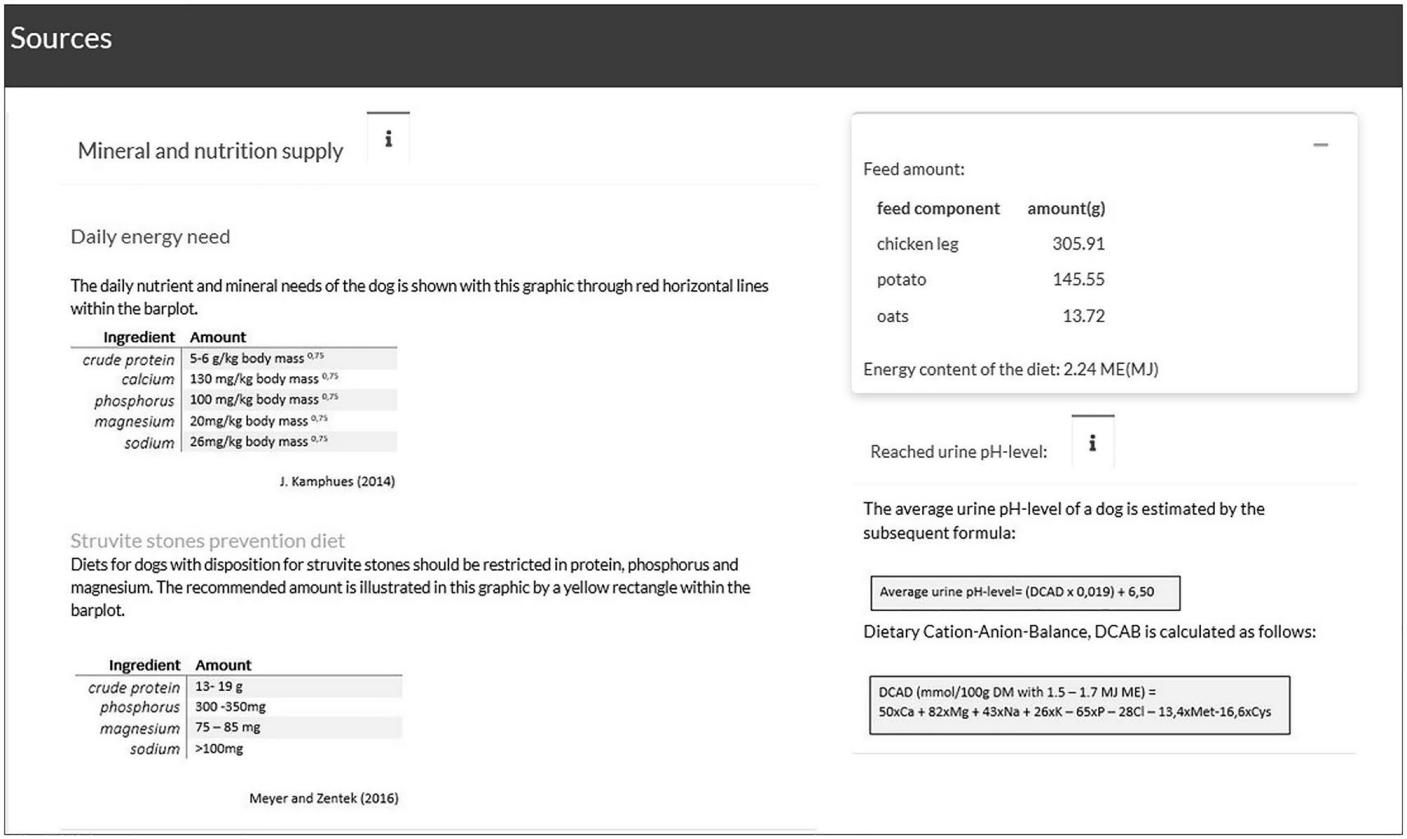
Screenshot of the panel with additional information regarding background information about the taught contents. This panel can be accessed by clicking on the information icon

A bar plot immediately displays the chosen components that are essential for a struvite stone formation. As the formation and dissolution of struvite stones in the urinary bladder depends on the urine pH, which in turn depends on the cation–anion balance, the urine pH is estimated and displayed based on the food composition at hand. Different coloured lines and rectangles, as depicted in Fig. 2, indicate whether the maintenance requirements of a dog referred to its bodyweight and the recommended mineral supply for struvite stone prophylaxis in the bladder are reached. A second tab located at the top of the application allows access to additional information (Fig. 3). These panels provide the student with background information and explanations on how to read the plot.

#### Food Science: Thermal Destruction of Microorganisms in Food Cans

The interactive graphic for the course food science was designed to simulate a practical scenario. The lecturer presents a case of an inadequate heating treatment of a food can (Supplementary Fig. 2). In cooperation with the lecturer, students can modify the heating curve by selecting the maximal core temperature and heating duration via slider elements. Instantly, the *F*-value, a sterilization value for food cans Ohlsson [24], is calculated and displayed beside the heating curve. Afterwards, students have four possible answers to determine the degree of durability for their theoretical cans. The durability depends on the *F*-value the students achieved with their different temperature and heating duration combinations.

### Access to Interactive Graphics

The raw files of all presented interactive graphics are freely available from the Github repository https://github.com/klausjung-hannover for any person interested in trying them out. A ReadMe file added to the repository adds information about the functionalities of the web applications, as well as instructions on how to install necessary R packages and to run the R scripts. At our own university, the interactive graphics are hosted within the university server, and students and lecturers can easily access the web application via a web browser at a faculty computer. We are working currently with our IT department to install a virtual machine so that ready-to-use interactive graphics are online accessible to everyone. We comment this current limitation in the “Discussion” section.

### Evaluation Study

We distributed a paper-based questionnaire to students who attended the three above described courses in the winter term 2019/2020. The participation in this study was voluntary and no personally identifiable information except for gender and age was captured. The data security office of our university approved the evaluation study, and all data were stored anonymously. The lecturers informed the students about the evaluation before the lectures and students completed the questionnaires afterwards. In total, *n* = 327 students returned filled questionnaires. In the zoology course, 89 students participated, all in their first study year. In the animal nutrition and the food technology course, 215 and 23 students, respectively, returned the questionnaires. In addition, *n* = 5 lecturers filled out questionnaires on their experience while teaching with the interactive tools. Student’s and lecturer’s questionnaires are available in the Supplementary Material. In the courses, German versions were used, but we present English translations as supplementary material. The contents of the questionnaires are described in the following.

#### Student’s Questionnaire

The student’s questionnaire included two demographic questions (sex and age) and nine questions regarding categorized into the subtopics:

- B) Interactive graphics in teaching and its handling
- C) Effects of the interactive graphic on the learning experience
- D) Digital media usage

Students were asked to rate on an ordinal scale from 1 (strongly disagree) to 5 (strongly agree) following statements.

- B1: I can imagine that the majority of students will handle easily interactive graphics.
- B2: I’d wish to have more interactive graphics in veterinary school.
- B3: I can image using this tool because it ´s intuitive.
- C4: I was previously familiar with the taught content.
- C5: I understood the teaching contents taught by the interactive graphic.
- C6: The interactivity of the graphic had a positive impact on my interest about the taught content.
- C7: The interactive graphic had no benefit to the course.
- D9: Digitalization is a chance to improve academic education.

Additionally, students were asked to provide information if and which other digital media they have been using for learning so far.

#### Lecturer’s Questionnaire

We also asked participating lecturers to complete a questionnaire after teaching with interactive graphics. The questionnaire had a similar design to the student’s questionnaire. Professors were asked to rate on an ordinal scale from 1 (strongly disagree) to 5 (strongly agree) the following statements:

- A1: I felt secure handling this teaching tool.
- A2: I can imagine that the majority of lecturers will handle easily this teaching tool.
- A3: I wish to have more interactive graphics in my classes.
- B4: In my opinion the interactive graphic provided no benefit to the course.
- B5: I feel like I could have reached more students by teaching with an interactive graphic.
- C8: Digitalization is a chance to improve academic education.

Furthermore, professors were asked (yes/no question) if they have used digital teaching tools in their classes before (question C6), and if the usage of interactive graphic had a positive effect on their opinion towards digital media usage in academic education (question C7). An additional open comment field allowed lecturers to express improvement wishes, comments or future design ideas.

### Data Analysis

Answer distributions of all questions were analysed graphically and descriptively, separately for each course and separated by gender and age. Effects of age and gender were assessed using the Mann–Whitney *U* test and Kendall’s correlation test, respectively. Correlations between the general usage of digital learning media (on a nominal scale) and several questions (with ordinal scale of answers) in the questionnaire were also studied using the Mann–Whitney *U* test. The significance level was fixed at alpha = 5%; in the case of multiple testing, the method of Bonferroni-Holm was used to adjust *p*-values. All analyses were performed using the statistic software R.

## Results

### Student’s Response

**Fig. 4:**
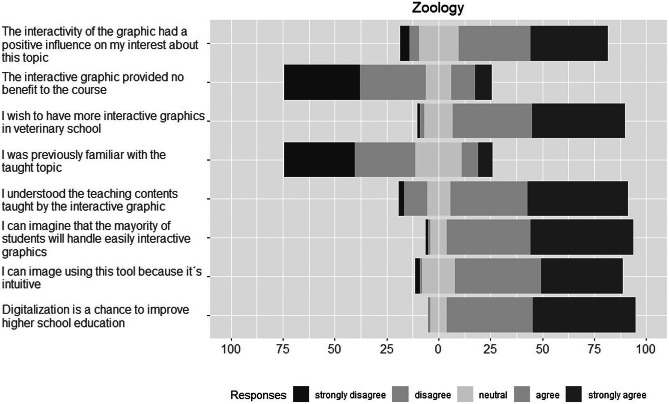
Answer distributions for Likert scale questions on the interactive graphic in the zoology course

**Fig. 5:**
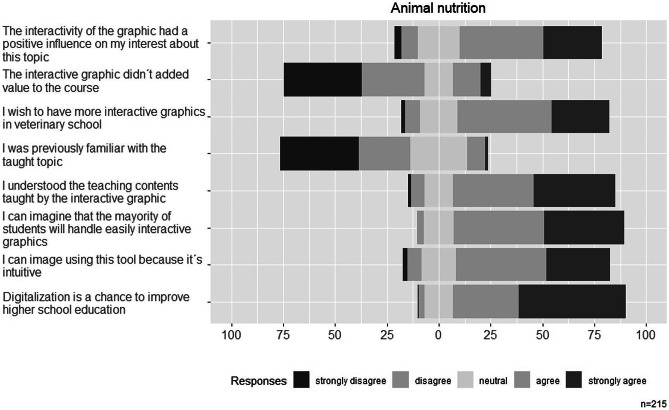
Answer distributions for Likert scale questions on the interactive graphic in the animal nutrition course

**Fig. 6:**
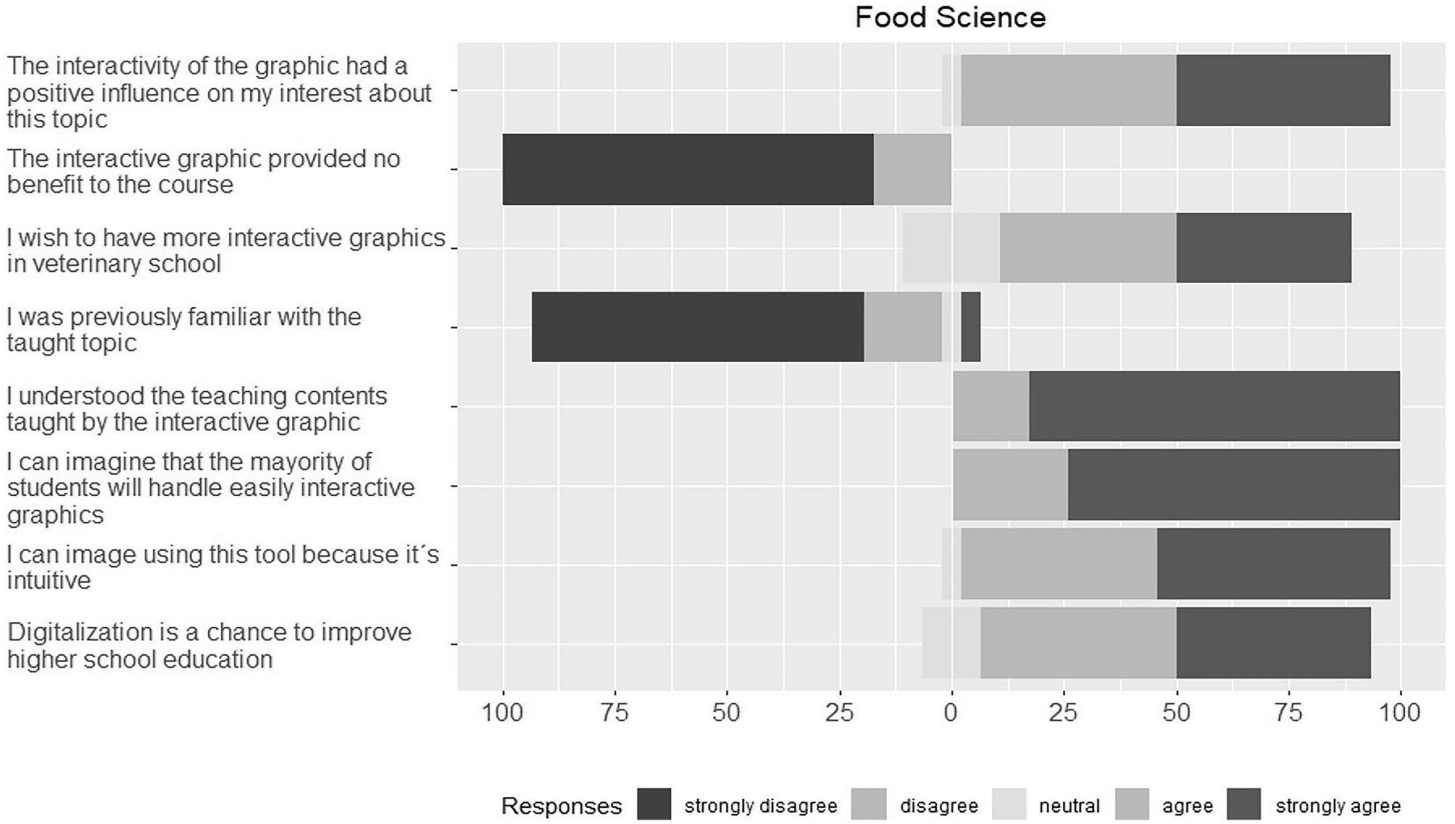
Answer distributions for Likert scale questions on the interactive graphic in the food science course

In general, we observed a similar answer distribution in all three describes courses (Figs. 4, 5, and 6). Absolute and relative frequencies for each answer are presented in the Supplementary Table S.T1–S.T3.

#### Demographic Results

In the zoology module, 72/89 (80.9%) participants were female and 16/89 (18%) were male. One student (1.1%) did not provide gender information. In the animal nutrition course, 185/215 (86.0%) were female and 26/215 (12.1%) were male, and 4 (1.9%) students did not answer the gender question. Finally, only women participated in the food science course (23/23). Thus, there was a similar gender distribution in the first two courses, but a different distribution in the last one. In general, large proportions of women are very typical amongst veterinary students. Age distribution (mean ± standard deviation years) was 20.3 ± 2.7 (minimum: 17, maximum: 29) for the zoology module, 24.5 ± 4.6 (minimum: 21, maximum: 57) for the food science module, and 21.9 ± 2.8 (minimum: 20, maximum: 29) in the animal nutrition course. Thus, the food science course was visited by older students compared to the two other courses.

#### Interactive Graphics in Teaching and Its Handling

In general, we found a high acceptance of interactive graphics as new teaching tool amongst all students in the three studied courses. Not less than 70% of students from fourth year agreed or strongly agreed to the statement “I’d wish to have more interactive graphics in veterinary school”. For students from first year, we found over 80% agreement with that statement. Regarding the handling of the web tool, most students were confident to be able to use easily the interactive tools themselves and estimate that other students will do so, too (over 70% agreement or strong agreement to answers of questions B1 and B3 in all three courses).

#### Effects of Interactive Graphics on Learning Experience

The previous knowledge about the taught topics was low amongst students in all courses. In the zoology course, 62.92% of the students strongly disagreed or disagreed to the statement “I was previously familiar with the taught topic”. In food science and animal nutrition lectures, 91.3% and 62.5% of the students, respectively, rejected (selected answers were “strongly disagree” or “disagree”) that statement, too. Despite the observation, students mainly agreed in all three courses to have understood the subject taught by this new teaching tool.

Regarding the effect of interactive graphics on the learning experience, the findings of the questionnaire yielded that students consider the interactivity of the graphic to have promoted their interest on the taught contents. In the food science course, 95.6% agreed or strongly agreed to this statement. Zoology and animal nutrition students agreed to 71.9% and 68.4%, respectively, to that statement. To correct the acquiesce tendency, we included a negatively worded statement. In all courses, students mostly disagreed or strongly disagreed to the assertion “The interactive graphic did not provide benefit to the course”.

#### Digital Media Usage as Learning Resource

The analysis of the student’s questionnaire shows evidence for a positive perception of digitalization in academic environment amongst veterinary medicine students. Most students considered digitalization as a chance to improve the academic education. Students in general seem to have a high acceptance for e-learning offered by the university and seek for other digital media to support their learning advances (Table 1). Besides online videos and forums, students use some other digital media, such as online libraries, multimedia websites and 3D-models. The number of students which do not use any digital learning material was low. Those students who do not use other digital learning media as a learning device tended to have a more neutral attitude regarding digitalization in higher education (Supplementary Tables S.T4–S.T5). More detailed, 30 (14%) of the students in the animal nutrition course that stated not to use other digital media, showed a significantly lower agreement to question D9 (“Digitalization is a chance to improve academic education”) compared to 183 (85%) of students who use other digital media for learning (adjusted *p* = 0.03). Likewise, the 3 (13%) of students in the food science course who stated not to use digital media showed also a significantly lower agreement to question D9 (adjusted *p* = 0.03).

**Table 1:** Numbers and percentages of students who use other digital media for learning as asked in the three courses

Course	*n*	e-learning at university	Videos	Online-forum	Other	None
Zoology	89	55 (62%)	51 (57%)	7 (8%)	1 (1%)	16 (17%)
Animal nutrition	215	101 (47%)	152 (71%)	29 (13%)	27 (13%)	30 (14%)
Food science	23	18 (78%)	16 (70%)	3 (13%)	3 (13%)	3 (13%)

#### Students’ Response by Gender and Age

We found no evidence for differences between females and males in their opinion towards interactive graphics and digital media usage. There was also no evidence for age effects in the distribution of answers in all three courses. We only observed a tendency for a difference (adjusted *p* = 0.06) in the negative worded question asked in the zoology: males found more often no added value by the new teaching tool (Table 2).

**Table 2:** Percentage distributions of response (grade of agreement: 1–5, ranging from strongly disagree to strongly agree) for Likert scale questions asked in the zoology course, separately for males and females. For question C7 (“no benefit to the course”), there is a tendency that males see less benefit of the interactive graphic than females (50% versus 12% of answers agree or strongly agree)

	**B1**		**B2**		**B3**		**C4**		**C5**		**C6**		**C7**		**D9**	
Gender	F	M	F	M	F	M	F	M	F	M	F	M	F	M	F	M
1	0	6	0	6	1	6	31	44	1	6	3	12	39	19	0	0
2	1	0	1	6	1	0	29	31	1	0	4	6	34	25	0	6
3	8	6	14	12	14	25	24	19	7	31	21	12	14	6	4	25
4	39	50	40	25	42	44	8	6	43	12	35	38	8	25	46	25
5	51	38	44	50	42	25	8	0	47	50	38	31	4	25	50	44
*n*	72	16	72	16	72	16	72	16	72	16	72	16	72	16	72	16
*p*	0.32		0.88		0.13		0.18		0.42		0.51		< 0.01		0.17	
*p*_Holm_	1.00		1.00		0.91		1.00		1.00		1.00		0.06		1.00	

### Rating of Interactive Graphics by the Lecturers

Four of the five lecturers stated that they can imagine that most teachers will easily handle the interactive graphics, two of them with strong agreement to this question (Table 3). One lecturer, however, did not strongly agree to this question. Asking the participating lecturers about their opinion regarding usefulness of interactive graphics, we found a general high acceptance. All participating lecturers felt to have reached more students through this new digital teaching tool.

**Table 3:** Answer distributions (grade of agreement: 1–5, ranging from strongly disagree to strongly agree) for *n* = 5 lecturers involved in teaching with interactive graphics

Question	Level of agreement						
	**1**	**2**		**3**	**4**		**5**
A1: I felt confident using this teaching tool	0	0		0	2		3
A2: I can imagine that the majority of teachers will handle easily this teaching tool	0	1		1	1		2
A3: I wish to have more interactive graphic in my classes	0	0		2	0		3
B4: The interactive graphic provided no benefit to the course	3	2		0	0		0
B5: I feel like I could have reached more students by teaching with interactive graphic	0	0		0	2		3
C8: Digitalization is a chance to improve higher school education	0	0		0	2		3
	**Yes**		**No**			**NA**	
C6: I have used previously digital teaching tools	1		4			0	
C7: The usage of interactive graphic had a positive effect on my opinion towards digital media in academic education	4		0			1	

Most lecturers (4/5) claimed to never have used digital media in their classes before, even though they commonly agreed or strongly agreed to consider digitalization as a chance to improve academic education.

The open field section in the lecturer’s questionnaire revealed little improvement wishes. One lecturer rated the interactive graphics to have a high potential and ideal environment to develop a case-based scenario but criticized the missing opportunity for students to self-manipulate the graphic in class. Other lecturers considered a stable internet access and high-quality beamers as important for a reliable usage of this teaching tool.

### Experiences and Recommendations for Further App Development

**Fig. 7:**
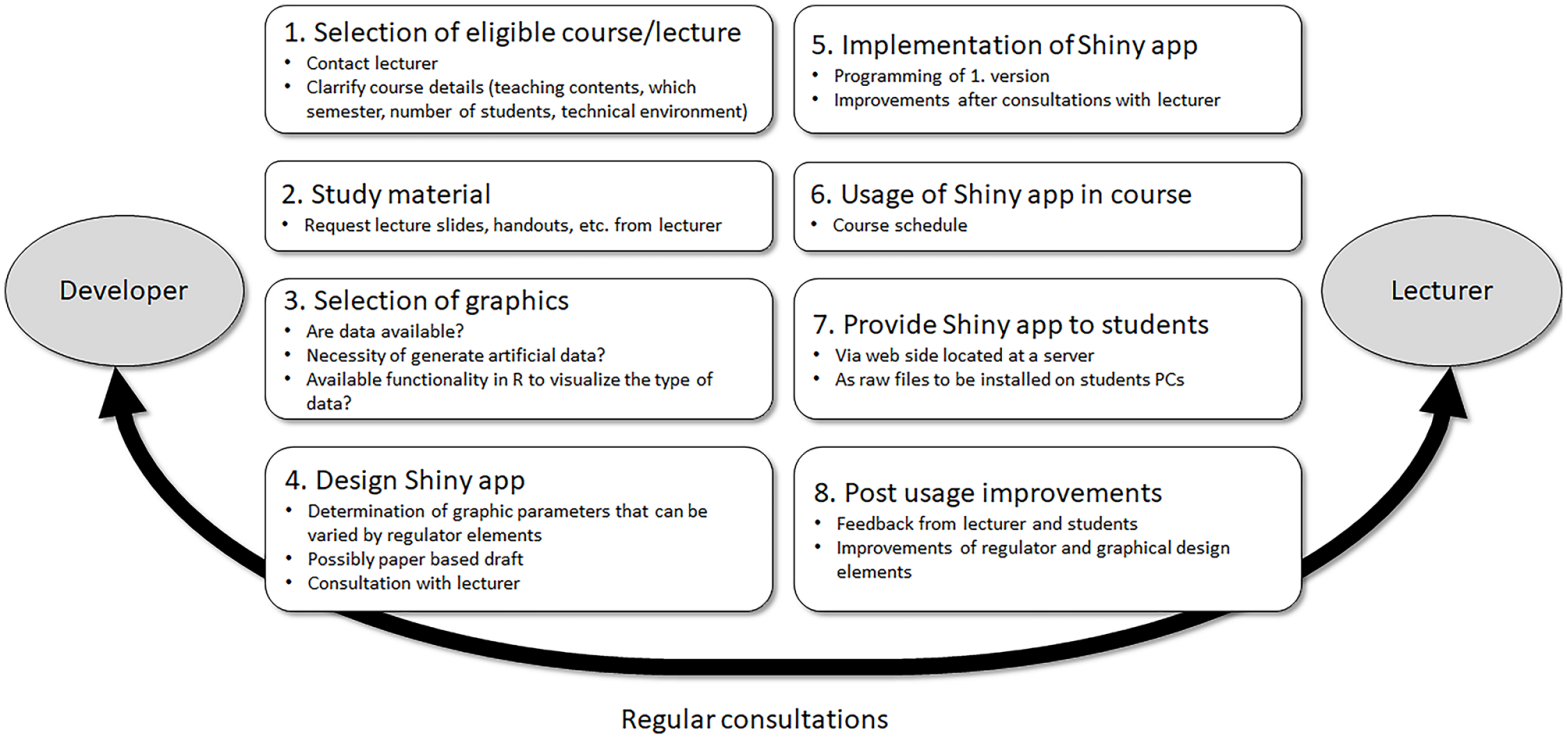
Recommended workflow of implementing an interactive Shiny app. For an optimal output, all steps require regular consultations between the developer of the app and the lecturer

During the development and usage of the three graphics, we collected our experiences regarding the individual steps and derived a workflow as recommendation for implementing and placing Shiny apps in a course (Fig. 7). As starting point, the developer group (e.g. as part of an e-learning facility) needs to select a course that appears eligible for a Shiny app (step 1). Either the developer may already know about a particular course and contact the lecturer, or the lecturer itself knows about the developers and asks for their help to develop an app for his or her course. In a first meeting, the developer should ask the lecturer for the course details such as the teaching contents, at which semester the course takes place, how many students typically attend the course, and which technical infrastructure is available in the course room. It may play a role whether only a PC and projector for the lecturer are available, or whether the course takes place in a computer lab with PCs for the students as well. The developers also must clarify whether the PCs have internet connection, or whether the app needs to be installed locally. To our experience, it is very helpful if the lecturer provides his current study material (lecture slides, handouts, etc.) so that the developer can screen for an appropriate graphic that can be implemented (steps 2 and 3). For the selection of a graphic, the developer must clarify whether the lecturer can provide own data or whether artificial data must be generated (e.g. by a random number generator following a particular statistical distribution or by mathematical formulae). Furthermore, functionality for analysis and visualization of specific data must be available in R. Eventually, special R packages need to be installed to run the app. As step 4, the design of the app should be drafted which can be done paper-based. We as developers also found it very helpful if the lecturer provides a sketch and his ideas on his own. Next, a first version of the app can be programmed by the developers, and followed by additional improved versions after consultations with the lecturer (step 5). When the lecturer is satisfied with a version, the app can be used in the course (step 6). After the course, the app should be made available to the students so that they can reflect the study contents on his own and in his own speed (step 7). The app can either be made available from a web page which runs from a university’s or a rented server. Taking feedback from students and lecturer, the app can be improved for the next semester, including optimization of regulator and graphical design elements (step 8). During all steps, we recommend regular consultations between the developer group and the lecturer.

## Discussion

In this pilot study, interactive graphics were implemented for selected courses from biological and medical disciplines, taught in veterinary education. Their general handling and their impact on students and educators were captured through questionnaires, and a general workflow for the development of interactive Shiny apps was derived. Summarizing the results of all three courses, 71% (233/326) of the students claimed that the interactivity of the graphics led to an increased interest for the presented study contents. Lecturers also agreed to have reached more students through teaching with interactive graphic.

Researchers in the field of educational psychology studied the role of interest in the learning process and revealed that interest is linked to motivation for learning and can also promote a deeper learning approach [25]. This is especially important in medical education, where students get instructed in a wide field of disciplines, such as diverse diseases, as well as basic natural science [26]. Obviously, not all disciplines can be of particular interest for the individual student and the workload is known to be quite high. Students facing a high workload eventually adopt a surface learning approach [27], where facts are just memorized and the meaning of the studied contents may not be entirely understood [28]. To conduce students into a deep learning approach, a “stimulating learning environment” must be promoted [29]. We believe to have contributed to such a stimulating learning environment by integrating these interactive teaching tools into our lectures. Our experiences in the app development and in the necessary collaboration between developers and lecturer also provide a basis for further research on the didactic effect of interactive graphics in veterinary education in comparison to teaching and learning with static graphics.

However, other studies comparing animated versus static images reveal inconsistent results regarding their effects on learning [30]. One common concern is the possible high informative content, which can lead to an overload. According to Hegarty, it is unclear whether all students have the metacognitive skills to effectively learn from interactive media [31]. Nevertheless, the here presented graphics were designed to be explored in collaboration with the professor during the lecture. In class, the teacher manipulates certain parameters through regulator elements while asking students to predict the outcome. This way students are encouraged to actively participate and think of the fundamental principles behind the presented graphic. After the class, students can manipulate the interactive graphics at their own pace by accessing a PC connected to the server of our university or by accessing the Shiny apps uploaded on the GitHub repository.

Considering that in total 76% of the students wish to discuss more topics with interactive graphics, it is worthwhile to analyse whether teachers in medical education would use interactive graphics for their classes. Even though the advances in online technology have reached medical education and diverse e-learning options are available [32], not all lecturers choose to implement them into their teaching [33]. Previous studies revealed that academic staff often remains attached to traditional ways of teaching, even though new technologies are fully integrated into the educational system Cuban [34, 35]. This matches the findings of our pilot study, which affirms that most of the participating professors (4/5) have never used digital teaching media in their class before. By involving the participating university professors actively into the development process of these teaching tools, we believe to have contributed positively not only to their general opinion towards digital media but also to their confidence in handling interactive graphics.

Past educational studies suggest that students respond positively to digital media in addition to traditional teaching. Therefore, one further aim of this work was to provide a positive handling experience and consequently stimulate digital media usage in class. After using interactive graphics for their class, 4/5 lecturers strongly agreed to the statement that this experience had a positive influence on their attitude towards digital teaching. Lecturers nowadays can currently choose out of a large collection of existing teaching applets and digital media resources in the web. However, they might not always find an existing teaching tool, which matches their individual needs and consequently might renounce to digital media for teaching.

For this study, we cooperated closely with the lecturers to build a teaching tool adopted to their individual wishes. The Shiny environment allows to display interactive graphics within a web browser and therefore can be easily integrated into lecturers’ presentations. Since its handling does not require any background knowledge in programming nor hardware or software installation, interactive graphics can be considered as user-friendly teaching tools. This assumption is confirmed by all lecturers involved (5/5), who claimed to have felt confident using interactive graphics during the class. When observing students’ perception towards the ease of use, in total, 78% (254/327) of the students agreed or strongly agreed to the statement that they would feel confident using interactive graphics by themselves. In total, 86% (279/326) of the students further believe their fellow students would do so, too. This positive attitude can be explained by the high acceptance for digital media and digitalization we yielded amongst the survey students. Only 49/327 of the students claimed to not use any digital media for learning. Those who do not support learning with digital resources also had a more neutral perception towards the effect of digitalization on higher education. Nevertheless, most students (86%; 280/327) and all lecturers (5/5) agreed that digitalization can improve higher education.

Still, a lack of training and programming skills can be an impediment for lecturers to use and develop further interactive graphics for their courses. Training teachers basic programming skills can be one possible way to spread interactive graphics through veterinary schools and other academic fields. Considering the time-consuming development process in addition to already high workload amongst teachers, the possibility of distributing already existing Shiny apps through free repositories, such as GitHub, gains special importance. Shiny apps can be shared as open educational resource which in return reduces the development efforts.

## Conclusion and Outlook

The findings of our pilot study revealed that the general handling of interactive graphics is not complicated, and that lecturers do not need specific training to use these tools in their courses. Furthermore, the veterinary students commonly agreed for the interactivity of the graphics to have a positive effect on their interest about the taught contents. This complies with the lecturer’s perception, to have reached more students by teaching with an interactive graphic. The lecturer’s positive perception towards this web tool is also a promising finding of this study in terms of promoting digital media usage at our university. Shiny applications facilitate data visualization and its modification in a user-friendly, visually appealing environment. We believe to have evidenced the potential of this web tool to create an enhanced learning and teaching experience in selected biomedical disciplines taught in veterinary education, where interactive graphics have rarely been integrated so far. Further work must be done, to examine if the integration of more interactive graphics in this field is feasible. In future, also apps may be developed that can contribute to scenario-based learning which is thought to let learners experience real-life scenarios in order to make them prepared for these scenarios [36]. This is not the case for all modules we tested in our study. While the food science and animal nutrition modules can be typical scenarios for a veterinarian, the zoology module only serves to explain a problem from natural sciences. Furthermore, our presented apps do not exploit the whole functionality of Shiny. Thus, much more powerful apps can be considered for future developments, including apps with images, videos and more interactive elements. As next step, we aim to conduct a randomized study to compare the didactic effect of interactive and static graphics in veterinary education.

## Supplementary Information

Below is the link to the electronic supplementary material.

Supplementary file1 (PPT 1191 KB)

## Data Availability

The data that support the findings of this study are openly available in Mendeley at https://data.mendeley.com/datasets/6th5cts43p/1 (https://doi.org/10.17632/6th5cts43p.1).

## Code Availability

R code for the Shiny applications is freely available at https://github.com/klausjung-hannover.
