# Supplementary material for: Interactive, Browser-Based Graphics to Visualize Complex Data in Education of Biomedical Sciences for Veterinary Students
Source: Med Sci Educ. 2022 Sep 22;32(6):1323–35. doi: 10.1007/s40670-022-01613-x (PMC9755394; doi:10.1007/s40670-022-01613-x)
Supplement: Supplementary file 1 — Supplementary file1 (PPT 1191 KB) [file 40670_2022_1613_MOESM1_ESM.ppt]

## Slide 1
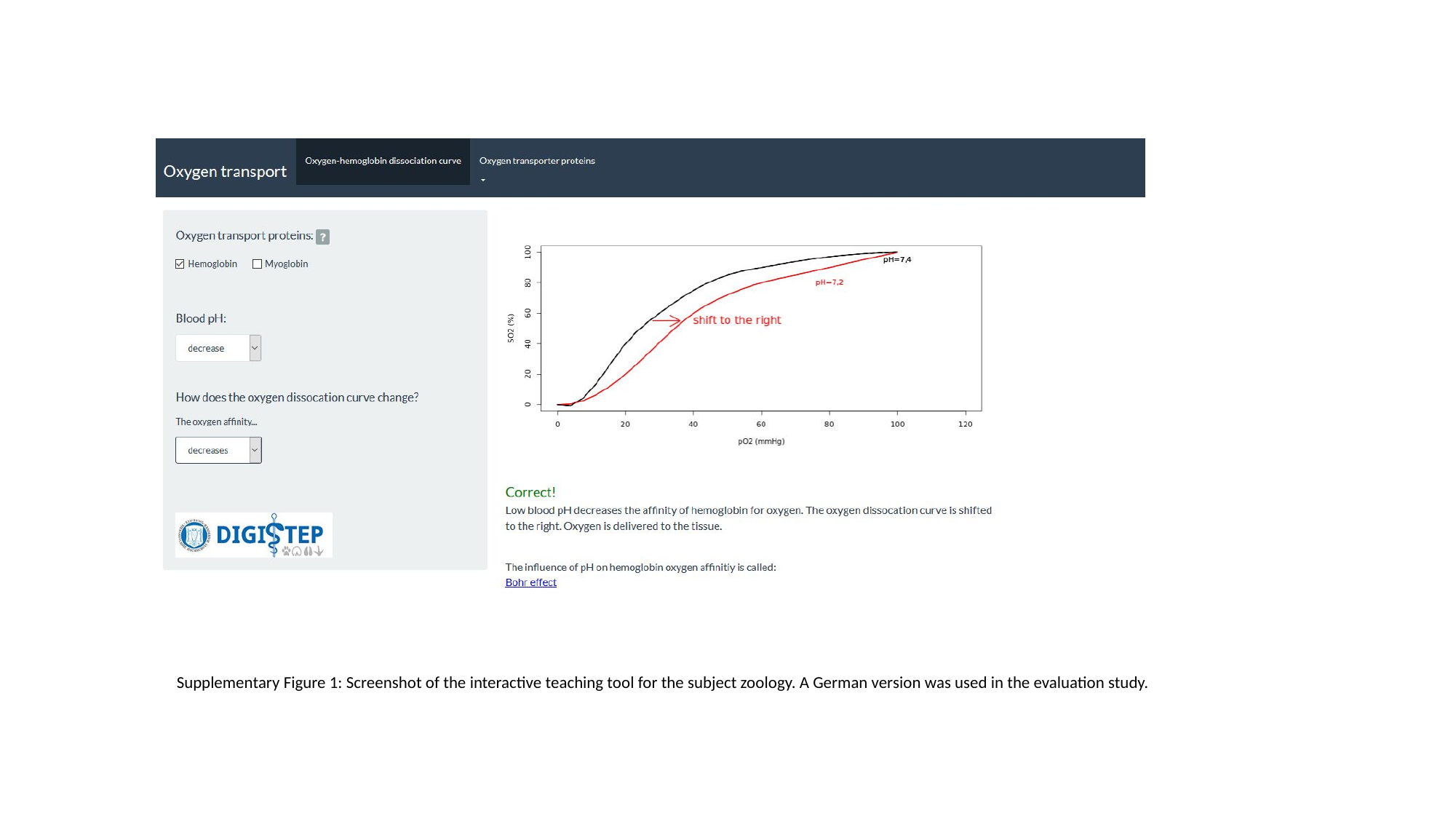

Supplementary Figure 1: Screenshot of the interactive teaching tool for the subject zoology. A German version was used in the evaluation study.

## Slide 2
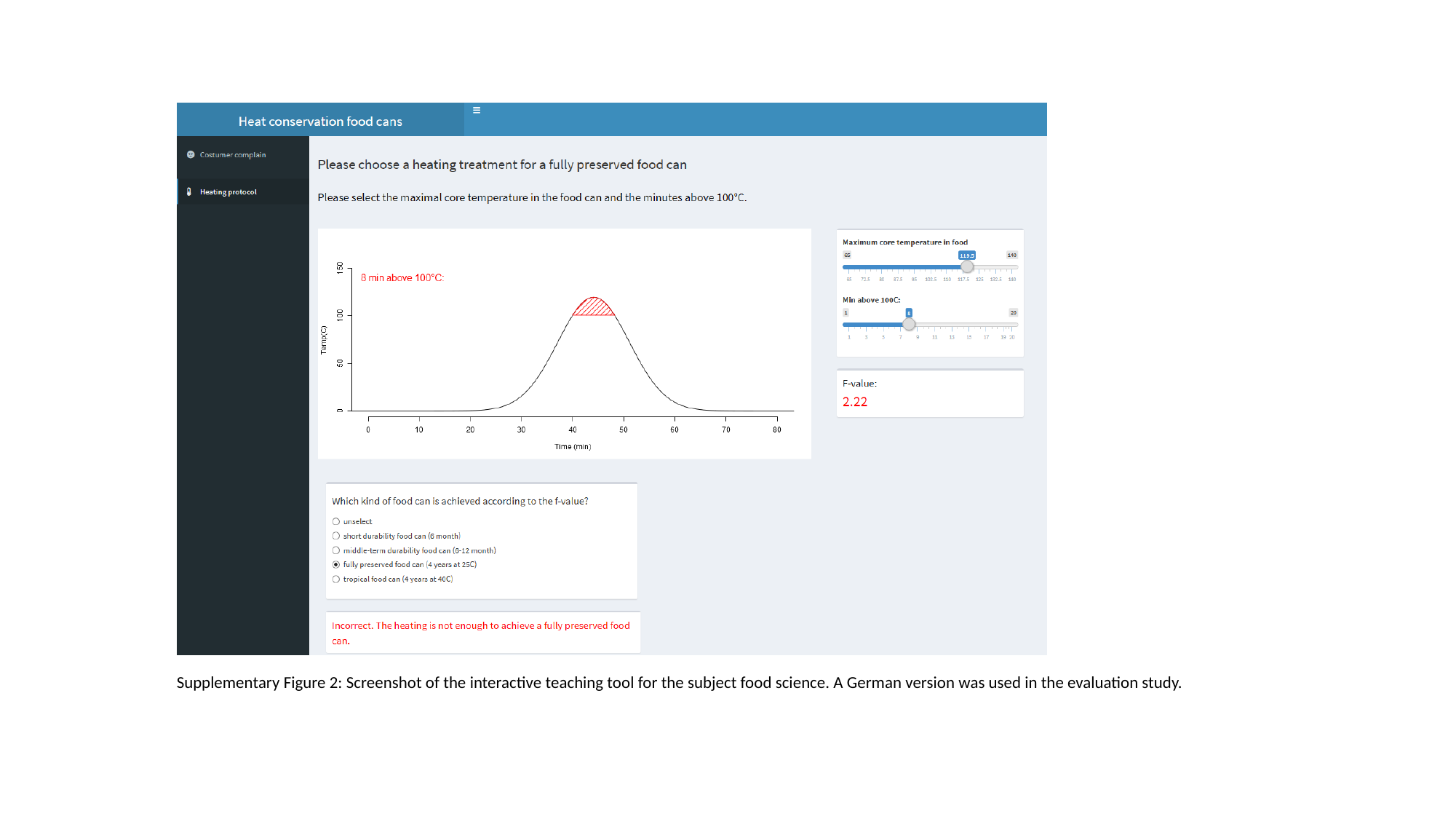

Supplementary Figure 2: Screenshot of the interactive teaching tool for the subject food science. A German version was used in the evaluation study.

## Slide 3
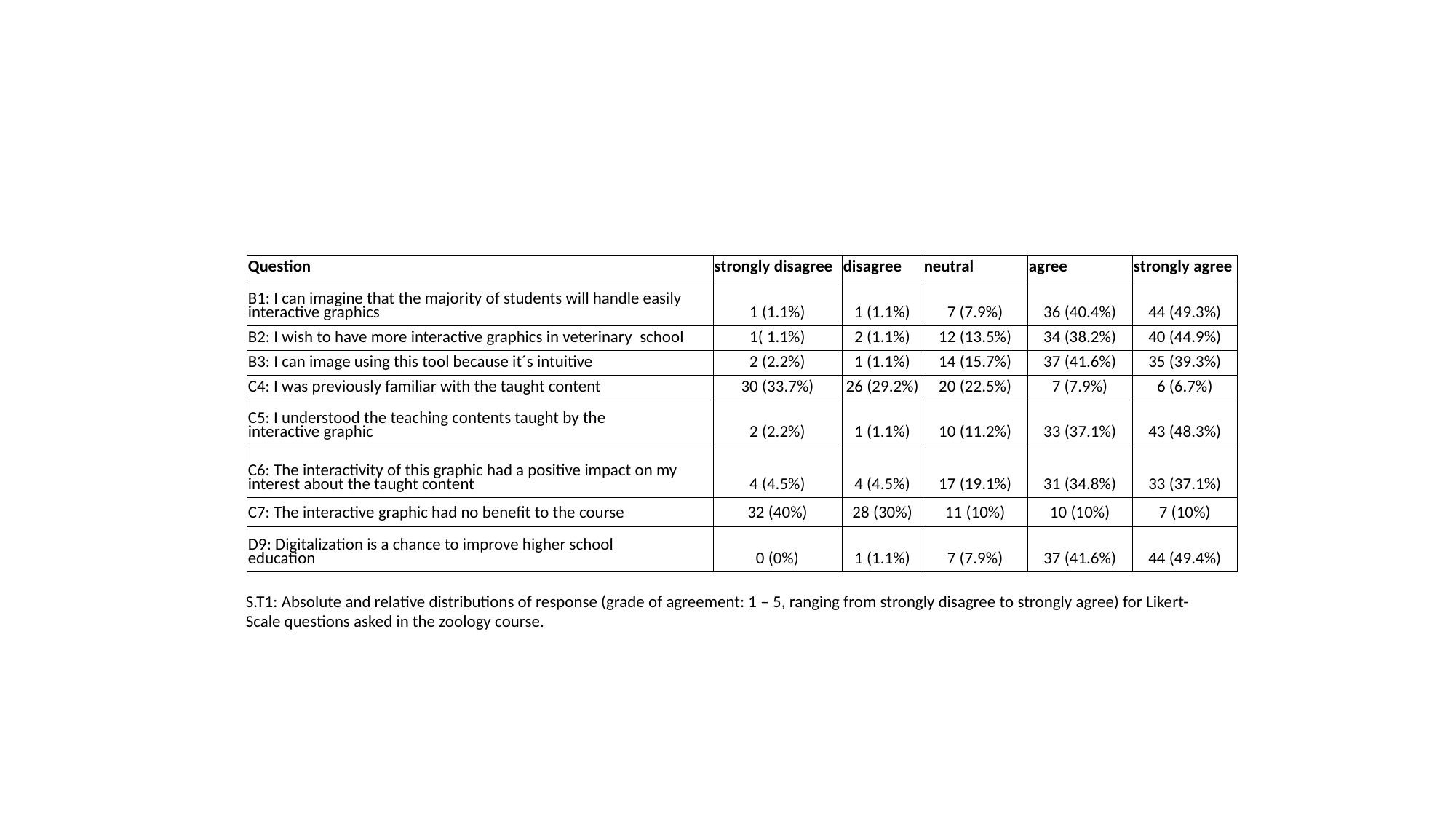

| Question | strongly disagree | disagree | neutral | agree | strongly agree |
| --- | --- | --- | --- | --- | --- |
| B1: I can imagine that the majority of students will handle easily interactive graphics | 1 (1.1%) | 1 (1.1%) | 7 (7.9%) | 36 (40.4%) | 44 (49.3%) |
| B2: I wish to have more interactive graphics in veterinary school | 1( 1.1%) | 2 (1.1%) | 12 (13.5%) | 34 (38.2%) | 40 (44.9%) |
| B3: I can image using this tool because it´s intuitive | 2 (2.2%) | 1 (1.1%) | 14 (15.7%) | 37 (41.6%) | 35 (39.3%) |
| C4: I was previously familiar with the taught content | 30 (33.7%) | 26 (29.2%) | 20 (22.5%) | 7 (7.9%) | 6 (6.7%) |
| C5: I understood the teaching contents taught by the interactive graphic | 2 (2.2%) | 1 (1.1%) | 10 (11.2%) | 33 (37.1%) | 43 (48.3%) |
| C6: The interactivity of this graphic had a positive impact on my interest about the taught content | 4 (4.5%) | 4 (4.5%) | 17 (19.1%) | 31 (34.8%) | 33 (37.1%) |
| C7: The interactive graphic had no benefit to the course | 32 (40%) | 28 (30%) | 11 (10%) | 10 (10%) | 7 (10%) |
| D9: Digitalization is a chance to improve higher school education | 0 (0%) | 1 (1.1%) | 7 (7.9%) | 37 (41.6%) | 44 (49.4%) |
S.T1: Absolute and relative distributions of response (grade of agreement: 1 – 5, ranging from strongly disagree to strongly agree) for Likert-Scale questions asked in the zoology course.

## Slide 4
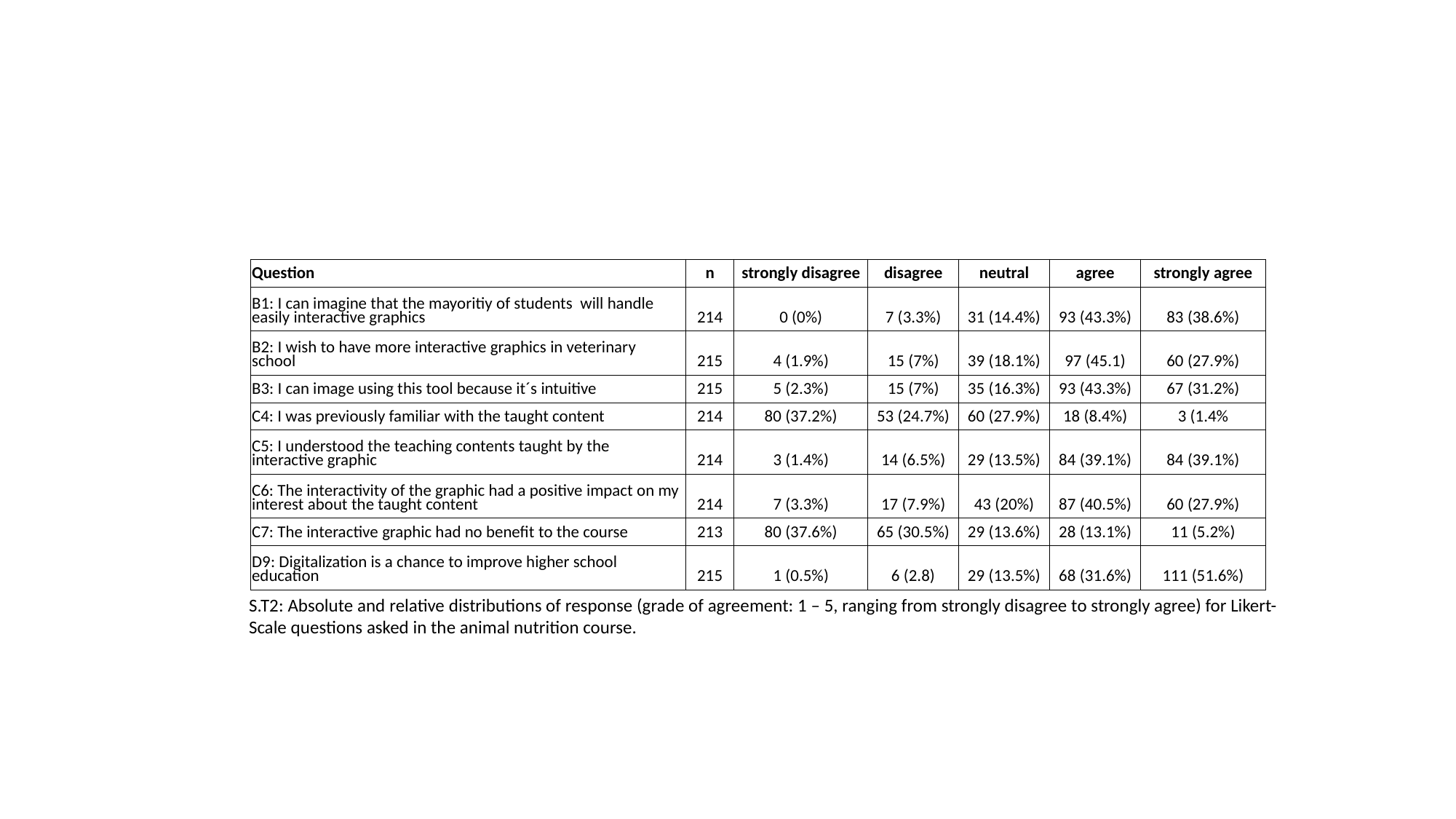

| Question | n | strongly disagree | disagree | neutral | agree | strongly agree |
| --- | --- | --- | --- | --- | --- | --- |
| B1: I can imagine that the mayoritiy of students will handle easily interactive graphics | 214 | 0 (0%) | 7 (3.3%) | 31 (14.4%) | 93 (43.3%) | 83 (38.6%) |
| B2: I wish to have more interactive graphics in veterinary school | 215 | 4 (1.9%) | 15 (7%) | 39 (18.1%) | 97 (45.1) | 60 (27.9%) |
| B3: I can image using this tool because it´s intuitive | 215 | 5 (2.3%) | 15 (7%) | 35 (16.3%) | 93 (43.3%) | 67 (31.2%) |
| C4: I was previously familiar with the taught content | 214 | 80 (37.2%) | 53 (24.7%) | 60 (27.9%) | 18 (8.4%) | 3 (1.4% |
| C5: I understood the teaching contents taught by the interactive graphic | 214 | 3 (1.4%) | 14 (6.5%) | 29 (13.5%) | 84 (39.1%) | 84 (39.1%) |
| C6: The interactivity of the graphic had a positive impact on my interest about the taught content | 214 | 7 (3.3%) | 17 (7.9%) | 43 (20%) | 87 (40.5%) | 60 (27.9%) |
| C7: The interactive graphic had no benefit to the course | 213 | 80 (37.6%) | 65 (30.5%) | 29 (13.6%) | 28 (13.1%) | 11 (5.2%) |
| D9: Digitalization is a chance to improve higher school education | 215 | 1 (0.5%) | 6 (2.8) | 29 (13.5%) | 68 (31.6%) | 111 (51.6%) |
S.T2: Absolute and relative distributions of response (grade of agreement: 1 – 5, ranging from strongly disagree to strongly agree) for Likert-Scale questions asked in the animal nutrition course.

## Slide 5
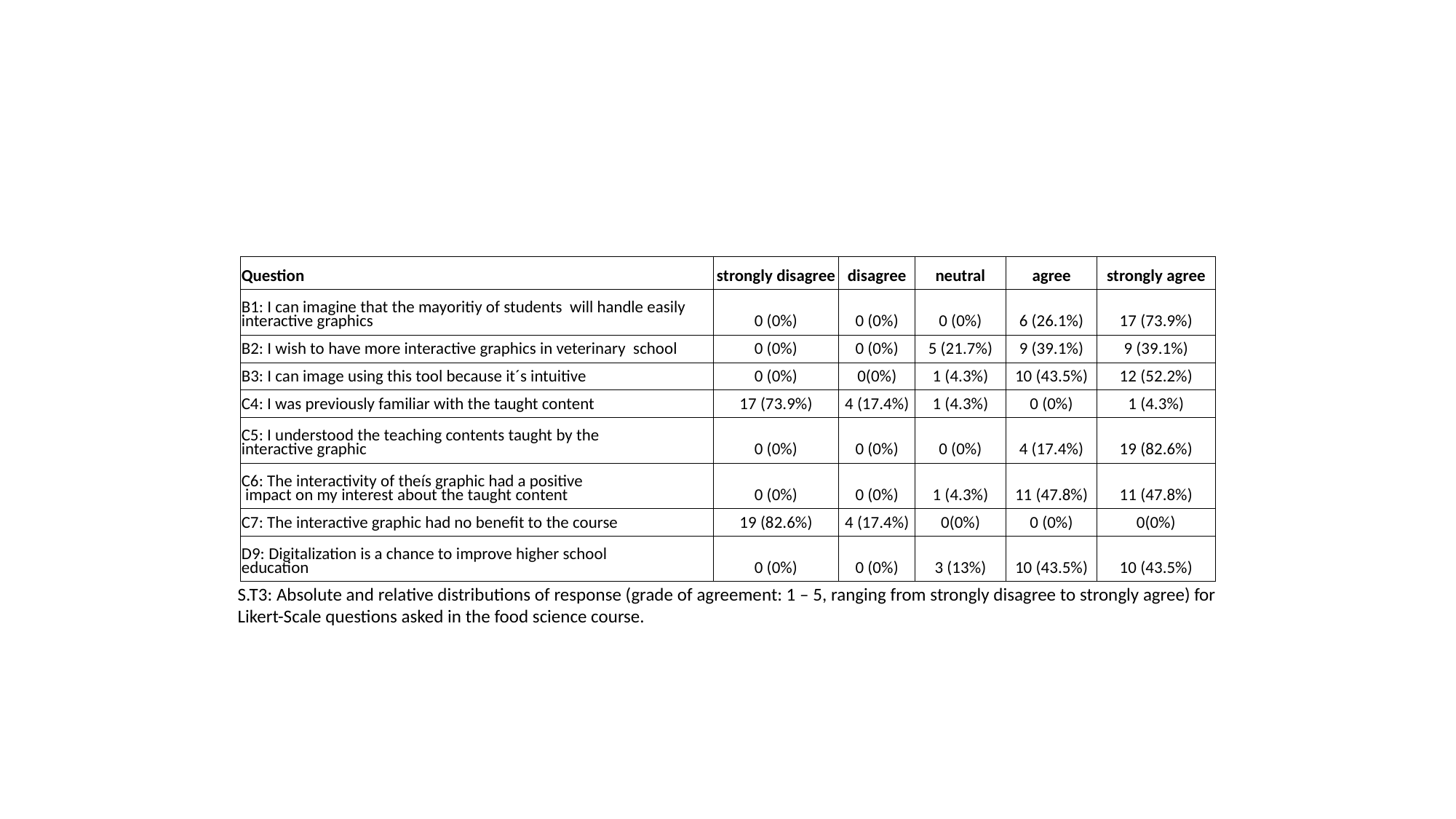

| Question | strongly disagree | disagree | neutral | agree | strongly agree |
| --- | --- | --- | --- | --- | --- |
| B1: I can imagine that the mayoritiy of students will handle easily interactive graphics | 0 (0%) | 0 (0%) | 0 (0%) | 6 (26.1%) | 17 (73.9%) |
| B2: I wish to have more interactive graphics in veterinary school | 0 (0%) | 0 (0%) | 5 (21.7%) | 9 (39.1%) | 9 (39.1%) |
| B3: I can image using this tool because it´s intuitive | 0 (0%) | 0(0%) | 1 (4.3%) | 10 (43.5%) | 12 (52.2%) |
| C4: I was previously familiar with the taught content | 17 (73.9%) | 4 (17.4%) | 1 (4.3%) | 0 (0%) | 1 (4.3%) |
| C5: I understood the teaching contents taught by the interactive graphic | 0 (0%) | 0 (0%) | 0 (0%) | 4 (17.4%) | 19 (82.6%) |
| C6: The interactivity of theís graphic had a positive impact on my interest about the taught content | 0 (0%) | 0 (0%) | 1 (4.3%) | 11 (47.8%) | 11 (47.8%) |
| C7: The interactive graphic had no benefit to the course | 19 (82.6%) | 4 (17.4%) | 0(0%) | 0 (0%) | 0(0%) |
| D9: Digitalization is a chance to improve higher school education | 0 (0%) | 0 (0%) | 3 (13%) | 10 (43.5%) | 10 (43.5%) |
S.T3: Absolute and relative distributions of response (grade of agreement: 1 – 5, ranging from strongly disagree to strongly agree) for Likert-Scale questions asked in the food science course.

## Slide 6
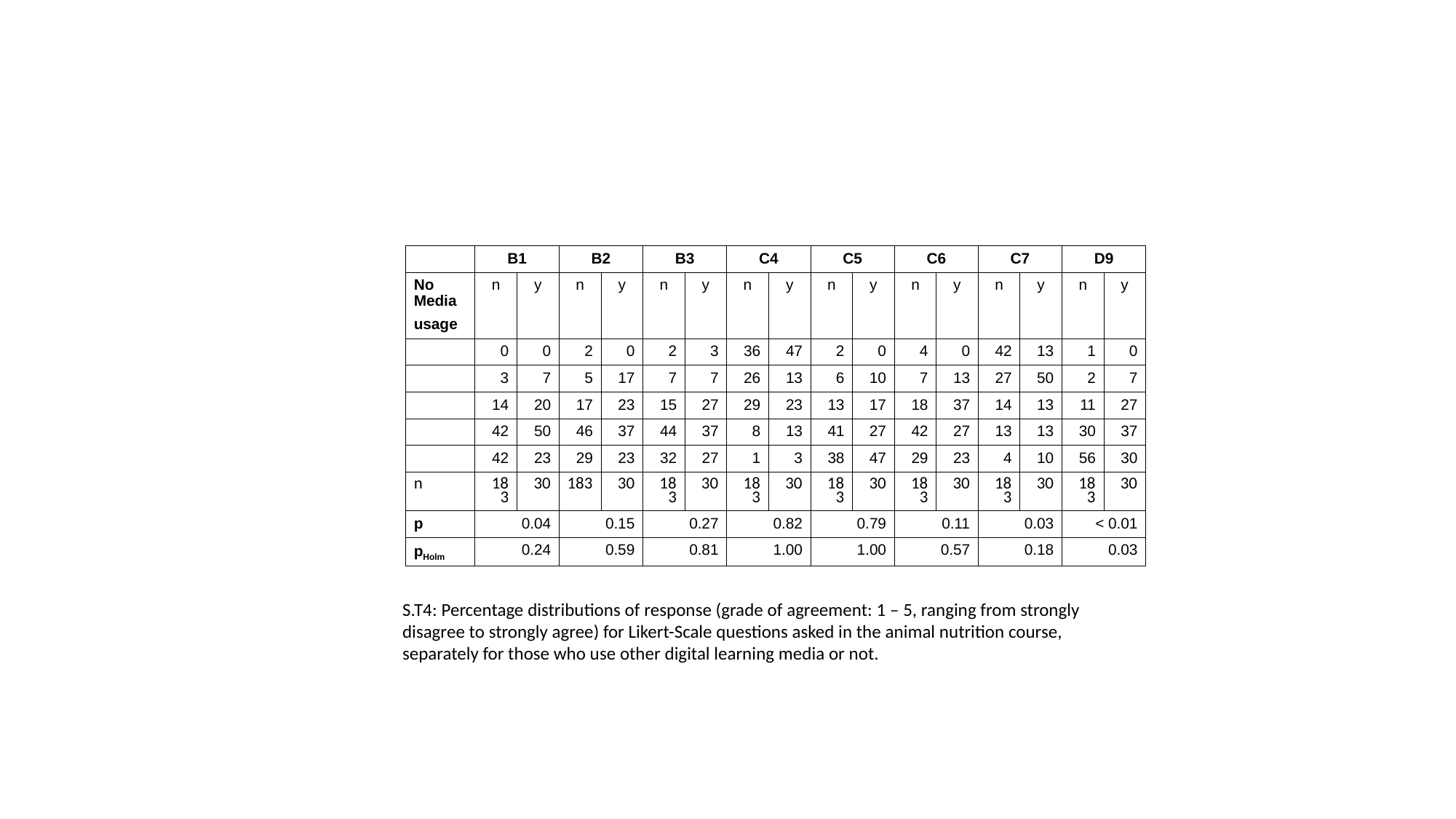

| | B1 | | B2 | | B3 | | C4 | | C5 | | C6 | | C7 | | D9 | |
| --- | --- | --- | --- | --- | --- | --- | --- | --- | --- | --- | --- | --- | --- | --- | --- | --- |
| No Media usage | n | y | n | y | n | y | n | y | n | y | n | y | n | y | n | y |
| | 0 | 0 | 2 | 0 | 2 | 3 | 36 | 47 | 2 | 0 | 4 | 0 | 42 | 13 | 1 | 0 |
| | 3 | 7 | 5 | 17 | 7 | 7 | 26 | 13 | 6 | 10 | 7 | 13 | 27 | 50 | 2 | 7 |
| | 14 | 20 | 17 | 23 | 15 | 27 | 29 | 23 | 13 | 17 | 18 | 37 | 14 | 13 | 11 | 27 |
| | 42 | 50 | 46 | 37 | 44 | 37 | 8 | 13 | 41 | 27 | 42 | 27 | 13 | 13 | 30 | 37 |
| | 42 | 23 | 29 | 23 | 32 | 27 | 1 | 3 | 38 | 47 | 29 | 23 | 4 | 10 | 56 | 30 |
| n | 183 | 30 | 183 | 30 | 183 | 30 | 183 | 30 | 183 | 30 | 183 | 30 | 183 | 30 | 183 | 30 |
| p | 0.04 | | 0.15 | | 0.27 | | 0.82 | | 0.79 | | 0.11 | | 0.03 | | < 0.01 | |
| pHolm | 0.24 | | 0.59 | | 0.81 | | 1.00 | | 1.00 | | 0.57 | | 0.18 | | 0.03 | |
S.T4: Percentage distributions of response (grade of agreement: 1 – 5, ranging from strongly disagree to strongly agree) for Likert-Scale questions asked in the animal nutrition course, separately for those who use other digital learning media or not.

## Slide 7
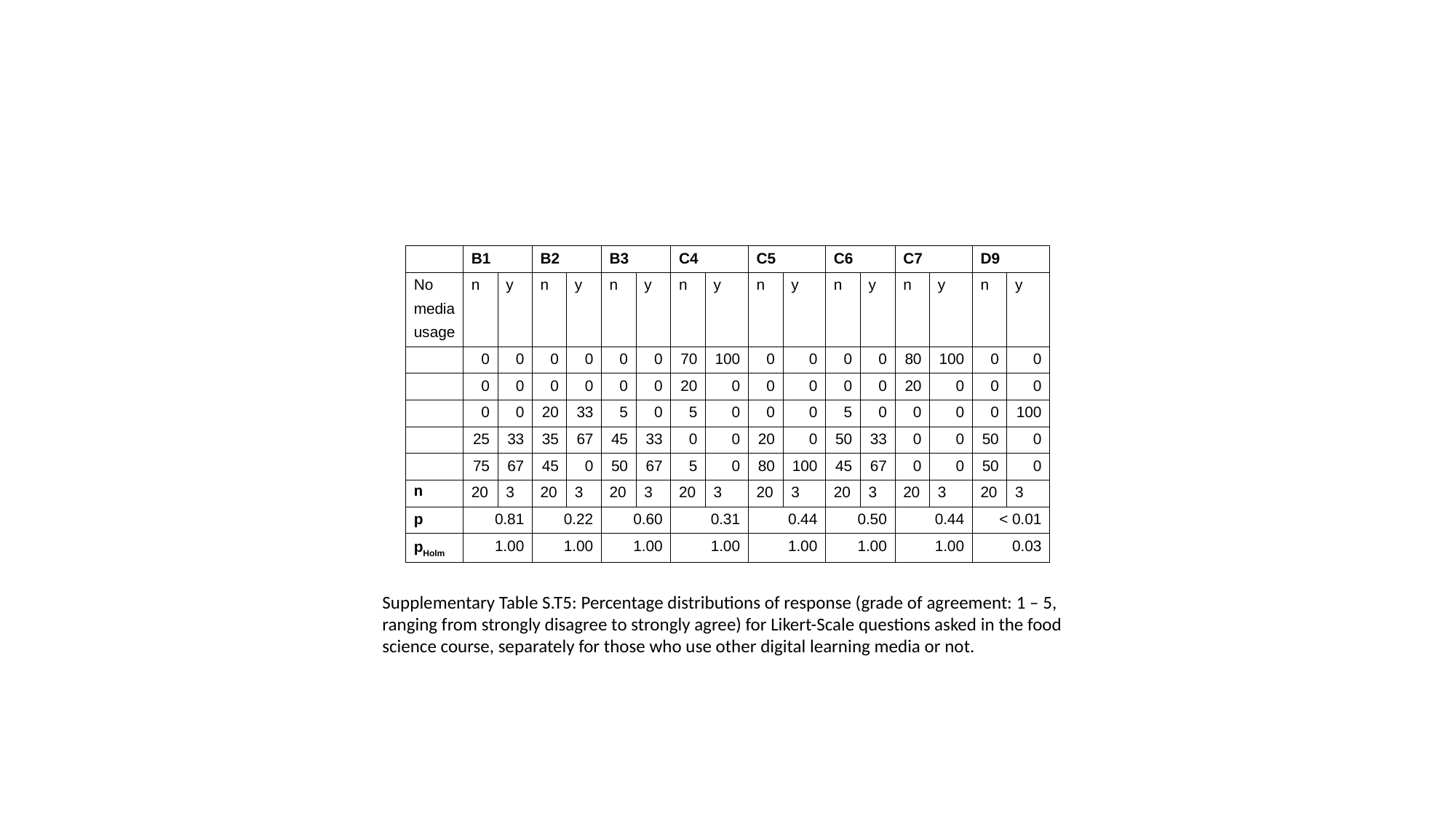

| | B1 | | B2 | | B3 | | C4 | | C5 | | C6 | | C7 | | D9 | |
| --- | --- | --- | --- | --- | --- | --- | --- | --- | --- | --- | --- | --- | --- | --- | --- | --- |
| No media usage | n | y | n | y | n | y | n | y | n | y | n | y | n | y | n | y |
| | 0 | 0 | 0 | 0 | 0 | 0 | 70 | 100 | 0 | 0 | 0 | 0 | 80 | 100 | 0 | 0 |
| | 0 | 0 | 0 | 0 | 0 | 0 | 20 | 0 | 0 | 0 | 0 | 0 | 20 | 0 | 0 | 0 |
| | 0 | 0 | 20 | 33 | 5 | 0 | 5 | 0 | 0 | 0 | 5 | 0 | 0 | 0 | 0 | 100 |
| | 25 | 33 | 35 | 67 | 45 | 33 | 0 | 0 | 20 | 0 | 50 | 33 | 0 | 0 | 50 | 0 |
| | 75 | 67 | 45 | 0 | 50 | 67 | 5 | 0 | 80 | 100 | 45 | 67 | 0 | 0 | 50 | 0 |
| n | 20 | 3 | 20 | 3 | 20 | 3 | 20 | 3 | 20 | 3 | 20 | 3 | 20 | 3 | 20 | 3 |
| p | 0.81 | | 0.22 | | 0.60 | | 0.31 | | 0.44 | | 0.50 | | 0.44 | | < 0.01 | |
| pHolm | 1.00 | | 1.00 | | 1.00 | | 1.00 | | 1.00 | | 1.00 | | 1.00 | | 0.03 | |
Supplementary Table S.T5: Percentage distributions of response (grade of agreement: 1 – 5, ranging from strongly disagree to strongly agree) for Likert-Scale questions asked in the food science course, separately for those who use other digital learning media or not.
